# New PET technologies – embracing progress and pushing the limits

**DOI:** 10.1007/s00259-021-05390-4

**Published:** 2021-06-03

**Authors:** Nicolas Aide, Charline Lasnon, Adam Kesner, Craig S Levin, Irene Buvat, Andrei Iagaru, Ken Hermann, Ramsey D Badawi, Simon R Cherry, Kevin M Bradley, Daniel R McGowan

**Affiliations:** 1 Nuclear medicine Department, University Hospital, Caen, France; 2 INSERM ANTICIPE, Normandie University, Caen, France; 3 François Baclesse Cancer Centre, Caen, France; 4 Department of Medical Physics, Memorial Sloan-Kettering Cancer Center, New York, NY USA; 5 Department of Radiology, Molecular Imaging Program at Stanford, Stanford University, Stanford, CA 94305 USA; 6 Institut Curie, Université PLS, Inserm, U1288 LITO, Orsay, France; 7 Department of Radiology, Division of Nuclear Medicine and Molecular Imaging, Stanford University, Stanford, CA 94305 USA; 8 Department of Nuclear Medicine, University of Duisburg-Essen and German Cancer Consortium (DKTK)-University Hospital Essen, Essen, Germany; 9 Departments of Radiology and Biomedical Engineering, University of California, Davis, CA USA; 10 Wales Research and Diagnostic PET Imaging Centre, Cardiff University, Cardiff, UK; 11 Radiation Physics and Protection, Churchill Hospital, Oxford University Hospitals NHS FT, Oxford, UK; 12 Department of Oncology, University of Oxford, Oxford, UK

## Introduction

Thanks to companies’ research and development processes, frequently involving fruitful partnerships with academic centres, and what could be acknowledged as welcome competition between PET vendors, new PET hardware and software technologies are regularly innovated and released as clinic-ready products. While some of these technological advancements have gained immediate acceptance from the nuclear medicine and medical physics communities, such as time of flight (ToF), others have not found support for translation. The case of point-spread-function (PSF) modelling within tomographic reconstruction, though unfortunately not unique, is a good representative example of an advanced reconstruction algorithm that has faced controversies, especially in the field of lymphoma imaging, despite numerous studies evaluating its diagnostic performance.

The lack of acceptance and integration of certain technologies may not necessarily be due to shortcomings in the technology. Successful translation is supported by several interacting phenomena and should be done with the aim of providing our patients with the highest diagnostic performance – and hopefully commensurate improved clinical management.

Many centres involved in the purchase of a PET system have observed a shift in the way the PET vendors compete with each other, no longer based solely on a technical superiority but also on business plans involving a significant decrease in injected dose and/or acquisition time. This reduction in injected dose (for obvious economic reasons, radiation safety and pressure of regulatory agencies) and in scan time (to reduce patient motion and discomfort but again also for economic reasons) sometimes jeopardizes the diagnostic performance achievable with modern PET systems.

This paper summarizes some research made by teams willing to champion and/or embrace new PET technologies and use them to reach the best diagnostic capabilities, even when performing fast imaging. Studies demonstrating the ability of PSF modelling, BPL reconstruction and SiPM PET with small-voxel reconstructions to improve detection of small cancer lesions will be summarized, and more recent advances such as motion correction, artificial-intelligence-based algorithms and total-body PET will be discussed in the real-life practice of busy PET centres. This review belongs to a two-part series of reviews published in EJNMMI addressing the pros and cons of new PET technologies. The complementary review by Julian MM Rogasch et al. [1] covers the cons.

## Advanced reconstruction algorithms: guilty until proven otherwise versus presumption of innocence

### The case of point-spread-function (PSF) modelling

PSF modelling, which is available from major PET vendors, has attracted considerable interest over the past 15 years. PSF modelling within tomographic reconstruction improves both spatial resolution and contrast recovery and reduces spatial noise, resulting in improved lesion detectability [2]. There is little room to question the fact that reconstruction algorithms including PSF modelling, alone or in combination with time-of-flight (ToF) capability, improve lesion detection: several studies have shown improvement in the diagnostic performance of ^18^F-FDG PET/CT in various cancers [3–7], especially for small cancer lesions. These studies also reported a significant increase in SUV metrics [8–10] and here lies the controversy on PSF modelling: the risk to produce artefacts, namely edge overshoot effect sometimes referred to as the Gibbs artefact, compromising the accuracy of quantitation in small lesions [11]. The use of ^18^F-FDG PET/CT in lymphoma patients has crystallized this controversy, with a focus on post-treatment evaluation using the Deauville criteria. Indeed, compared to standard OSEM reconstruction, PSF modelling significantly increases SUV in small tumour lesions but moderately impacts SUV metrics in big lesions and large reference organs such as the liver; this is what is required for improved lesion detectability. However, Deauville score (DS) uses reference organs to discriminate between responders (residual tumour uptake > liver) and non-responders (residual tumour uptake < liver) in non-Hodgkin lymphoma (NHL), and PSF modelling within reconstruction could in theory systematically increase DS.

Authors in this controversies article published a study involving 126 consecutive patients with DLBCL receiving first-line immunochemotherapy and comparing DS assessed on images produced with unfiltered PSF reconstruction versus European Association of Nuclear Medicine Research limited (EARL)-compliant OSEM [12]. Their study showed that major discordances (i.e. responders (DS 1–3) versus non-responders (DS4 and 5)) occurred only in 5% of interim PET (iPET) and in 3.2% of end-of-treatment PET (EoT PET) and, most importantly, that no difference in terms of risk stratification (using progression-free and overall survival) was observed between PSF and OSEM images. Following this publication, a letter from Boellaard et al. [13], focusing on patients quoted DS3 on OSEM images from the Enilorac et al. series [12] upstaged to DS4 because of PSF modelling within the reconstruction, stated that the occurrence of this shift from DS3 to DS4 (4/22, 18% for iPET and 3/18, 13% for EoT PET) was a strong argument against altering the status quo in multicentre trials, with no comment with regard to the use of PSF in clinical routine.

**Fig. 1:**
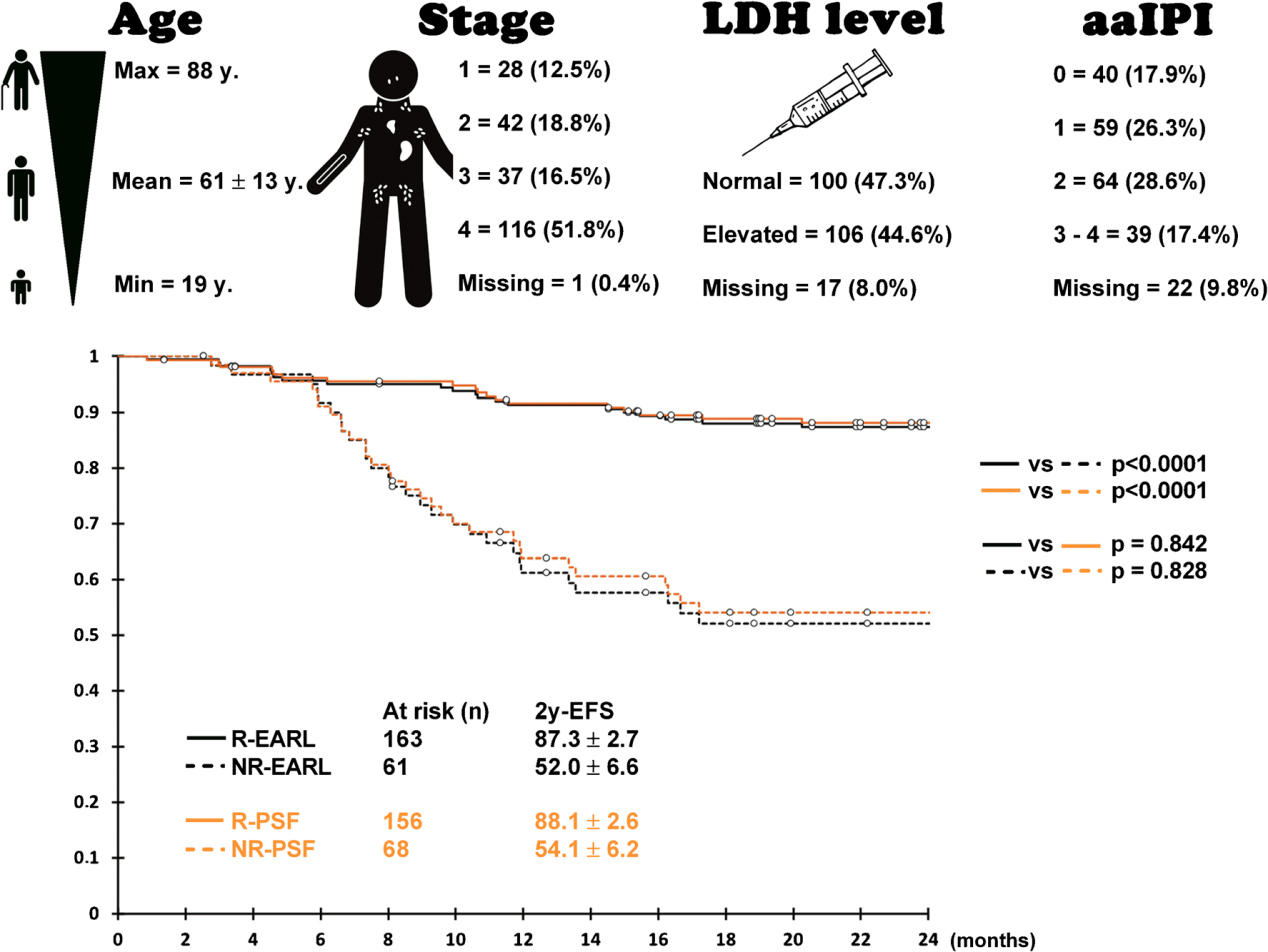
PSF does not affect Deauville scoring compared to former generation PET system: Kaplan-Meier survival curves displaying 2-year event-free survival (EFS) according to Deauville score (R, responders (DS 1–3); NR, non-responders (DS 4&5)) in diffuse large B cell lymphoma patients receiving first-line treatment for unfiltered images reconstructed with PSF modelling and EARL compliant images mimicking former generation PET systems. The upper panel describes patients’ characteristics: from right to left age, Ann Arbor stage, LDH blood level and age-adapted international prognostic index (aaIPI)

In order to make the case for the use of PSF modelling reconstruction for DS, we herein report unpublished data in an expanded series of 224 consecutive DLBCL patients receiving first-line immunochemotherapy. The exact same methodology as that used for the Enilorac et al. series was applied, but this time we focused on DS3 patients with conventional or EARL-compliant reconstruction shifted to DS4 because of PSF modelling reconstruction (*n* = 8/224, 3.6%), as the outcome of these patients with discordant findings are of immense value to better understand the impact of clinical gain or detrimental effect of PSF modelling. The PET system used in this series being EARL-accredited since 2005, and as such committed to use EARL-compliant SUVs for quantitative purposes [14] including DS, the patient’s management and related survival data were based on EARL reconstruction. As can be seen from Fig. 1, there is no statistical difference in event-free survival (EFS) according to DS responders or non-responders when comparing EARL-compliant reconstruction to one using PSF modelling.

### The use of Bayesian penalized likelihood (BPL) reconstruction

In addition to incorporating PSF modelling into the image reconstruction process, regularization can be integrated into the iterative image reconstruction process to enable more iterations without excessive noise amplification with the goal of getting closer to convergence to the maximum likelihood solution. This yields better contrast recovery without amplifying noise in the reconstructed images, aiding the detection of small abnormalities and improving quantification for patient benefit.

At the forefront of these regularization methods, the Bayesian penalized likelihood (BPL) reconstruction technique (which may also include PSF modelling) is increasingly being used. However, owing to the appearance of highly localized regions of count density and the associated SUV increases that result, some centres have not embraced BPL reconstruction for all clinical indications, particularly lymphoma [15, 16]. Currently, at least one PET vendor has implemented BPL reconstruction [17], although the basic formulation of BPL for iterative PET image reconstruction has been known since the 1980s [18]. The method employs a regularization term during the iterative reconstruction process which minimizes image noise at each update, enabling more iterations so that the image can be reconstructed towards effective convergence. Without such regularization, the number of iterations has to be limited at the expense of the recovery of the high-frequency components of the signal, to ensure adequate image quality without excess noise amplification.

**Fig. 2:**
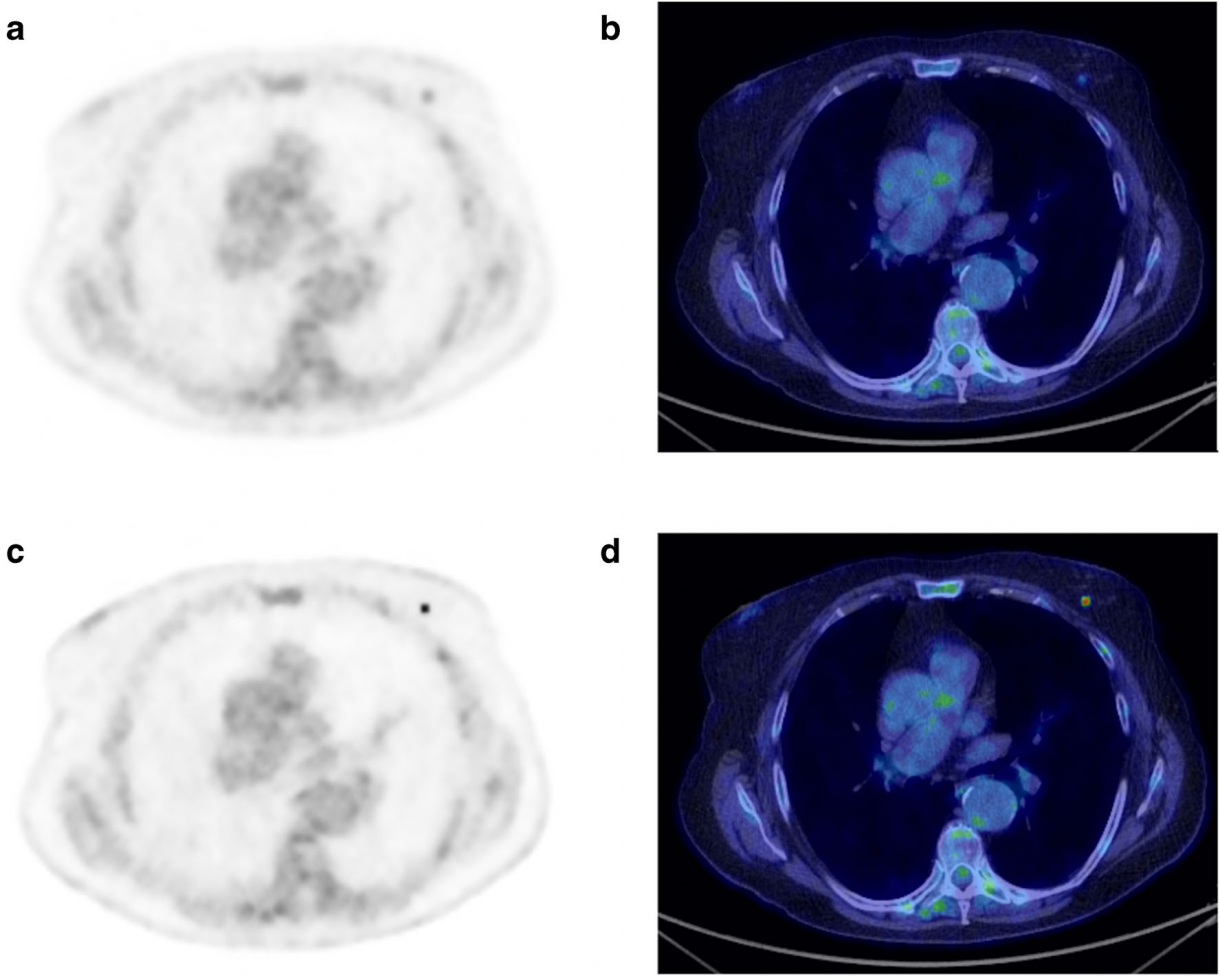
Incidentally detected tiny, ^18^F-FDG avid breast nodule in a 70-year-old patient. Triple assessment (mammogram, ultrasound and breast examination) following PET was negative, but 3 months later a small (sub-centimetre) ductal cell breast carcinoma was detected and cured. SUV_max_ 1.8 on OSEM (**a** and **b**) and SUV_max_ 5.0 on BPL (**c** and **d**). PET images on an SUV scale 0–6

Using BPL reconstruction in clinical studies enables improved lesion detection, and this has been shown in a wide variety of studies, for example, for ^18^F-FDG in lung nodules [17, 19], mediastinal nodes [20] and liver metastases [21]. PET imaging with other tracers also benefits from BPL reconstruction [17], for example, ^68^Ga-PSMA [22, 23], ^68^Ga-DOTATOC [24], ^68^Ga-RM2 [25], ^90^Y-SIRT [26, 27], ^18^F-PSMA [28], ^18^F-NaF [29], ^68^Ga-citrate [30], ^18^F-FACBC [31], ^13^N-NH3 [32], ^11^C-acetate [24] and ^89^Zr-immuno-PET [33]. In addition, and perhaps most importantly, BPL is particularly advantageous in patients with high BMI [34, 35], because they usually have the greatest background image noise where both the detection and quantification of small abnormalities are most problematic. These potential benefits of BPL are vitally important especially in oncology where the detection of small lesions, such as early metastases, is essential and can drastically change patient management. An example image showing OSEM and BPL reconstruction of the same patient is shown in Fig. 2 for a sub-centimetre breast nodule, clearly showing the stated benefits.

Improvements in reconstructed image quality with changes in lesion SUV, particularly when combined with reduced noise, can make scan interpretation and reporting more difficult at first. As lesion SUV values increase with BPL reconstruction, previously used threshold criteria should be updated (as they have before when technology evolves).

Considering ^18^F-FDG scans, small metabolically active structures become far more visible; the aortic wall is seen separate to blood pool even in young, normal individuals; adrenal glands become far more conspicuous; and the spinal cord becomes prominent. More difficult: small ‘reactive nodes’ for instance in the neck can be prominent, and the pattern of small, symmetric ^18^F-FDG avid bilateral hila and mediastinal nodes, often considered a ‘sarcoid-like’ reaction to malignancy, becomes more conspicuous. However, with experience, these issues become easy to recognize as normality, or as benign patterns, rather than causing false positives; and, as with any new technology that shows marked differences from previous versions, we advocate training and understanding of BPL reconstruction’s effects on imaging studies as key in the path towards widespread adoption [36]. The enhanced visualization of small foci of uptake in the image are categorically not ‘false positives’; in fact, even the inclusion of both PSF modelling and BPL in image reconstruction still results in underestimation of true activity in small foci; however, we contend that the enhanced observations with PSF modelling and BPL reconstruction are a step forward and nearer to achieving ‘phantom truth’ [37].

## Advanced PET detectors

### Pushing the limit of detectability

**Fig. 3:**
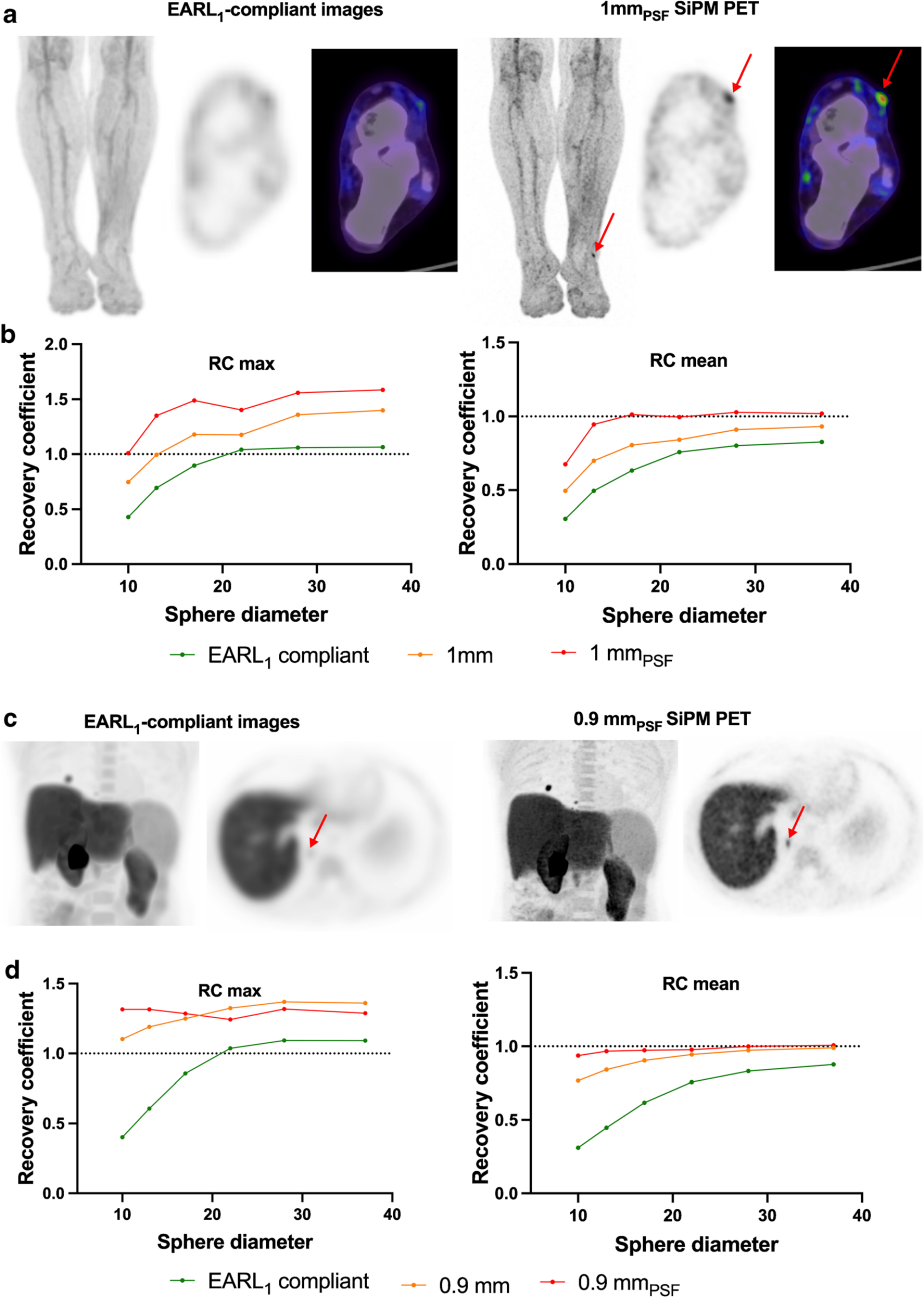
SiPM PET pushes the limits of detection of small lesions and is close to absolute quantitation. Panel **a** displays ^18^F-FDG images acquired on a Phillips Vereos PET system (PSF modelling and ToF enabled, small-voxel reconstruction (1 mm)). PET data were also reconstructed as per the EARL harmonizing standard, by applying a 7.2-mm FWHM Gaussian filter. This patient was referred for restaging of melanoma of the lower left limb and only SiPM PET was able to detect a single in-transit metastasis (red arrow). Panel **c** displays ^18^F-fluorocholine images acquired on a Siemens Vision PET system (PSF modelling and ToF enabled, small-voxel reconstruction (0.9 mm)). PET data were also reconstructed as per the EARL harmonizing standard, by applying a 9-mm Gaussian filter. The patient was referred for restaging after liver graft for hepatocellular carcinoma. A tiny lung metastasis (red arrow) was detectable only on SiPM PET. Images have been scaled on the same maximum value. Panels **b** and **d** show recovery coefficients using the same reconstruction parameters on a NEMA NU2 phantom: quantitation for mean values using small-voxel PSF images is close to the phantom truth except for the smallest sphere

More recently, silicon photomultiplier (SiPM) technology has become available from the major PET vendors. One of these systems, the Philips Vereos, is a PET camera featuring small SiPMs with a 1-to-1 crystal coupling, enabling small-voxel (1 mm) reconstruction [38]. This technology improves the detection of small lesions, as shown recently in a study evaluating SiPM ^18^F-FDG PET/CT with small-voxel reconstruction for detecting in-transit metastases in melanoma patients with a primary lesion located on the upper or lower limbs, in comparison with standard reconstruction and EARL-compliant reconstruction mimicking former generation PET systems [39]. The use of fine matrix reconstruction (either 1 mm or 1 mm_PSF_) led to an increase in tumour/background (a 2.84-fold increase in the case of 1 mm_PSF_ reconstruction) resulting in better sensitivity and specificity, the best compromise being the 1-mm reconstruction with a sensitivity and specificity of 92% and 94%, compared to 73% and 91% for EARL-compliant reconstruction, respectively. Figure 3 illustrates these findings and demonstrates, in line with the usefulness of PSF modelling and BPL reconstructions discussed above, that using state-of-the-art PET systems at the maximum of their capabilities not only significantly improves diagnostic performance but also provides quantification closer to phantom truth for pertinent sized lesions. The NEMA recovery coefficient curve is essentially at 100% for all but the smallest sphere using 1-mm voxel reconstruction with PSF modelling. As expected, the SUV_max_ is higher than 100%, this metric based on the value from a single voxel hence is significantly impacted by noise (although very consistent between reporters and software due to its simplicity). With older PET reconstructions, the SUV_mean_ was underestimated with the SUV_max_ values actually being closer to the real SUV_mean_, and now with modern PET systems, the SUV_mean_ becomes far closer to the actual value within the patient.

### Standard vs SiPM PET

**Fig. 4:**
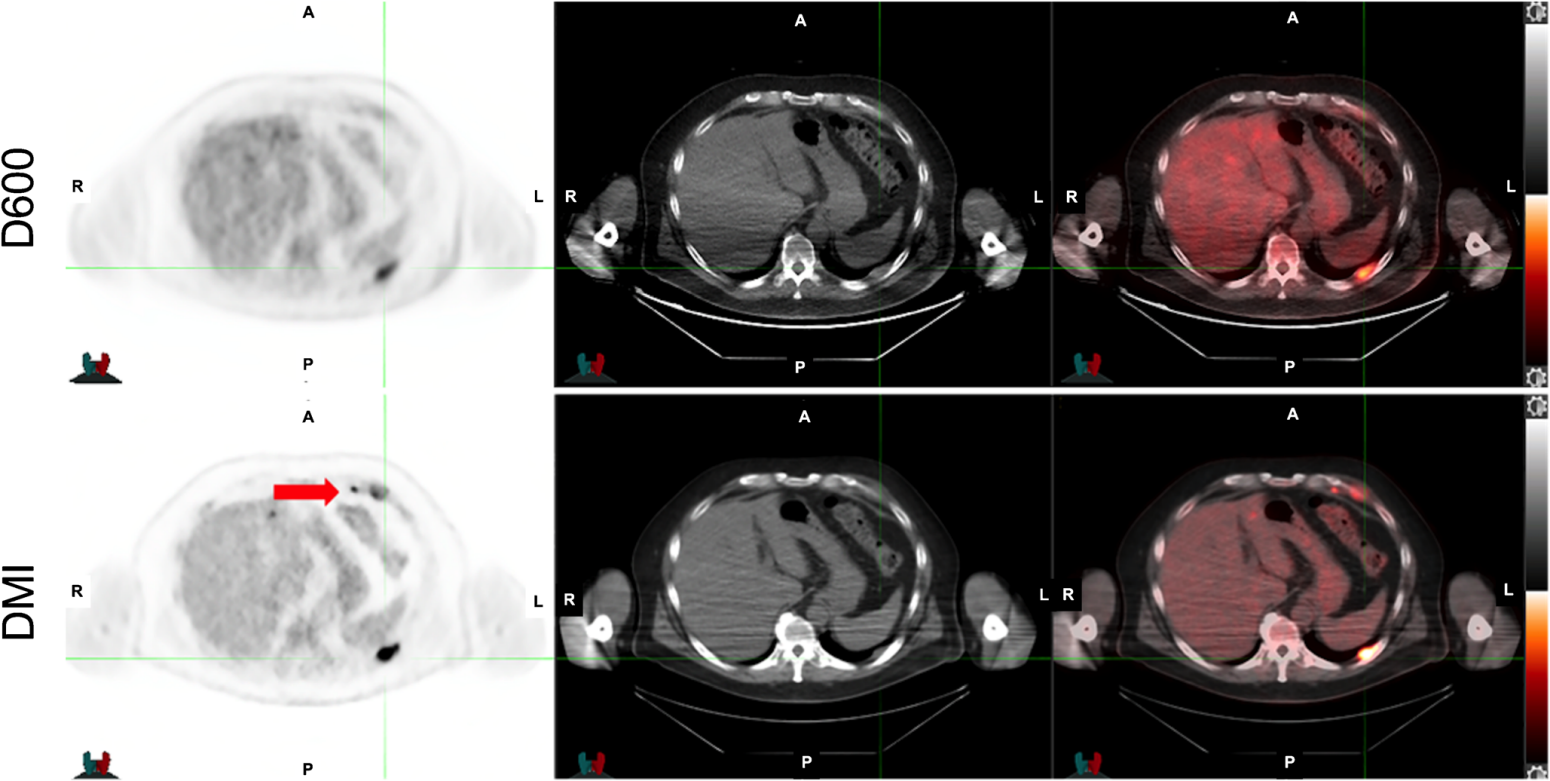
SiPM PET using BPL reconstruction versus PMT PET. A 57-year-old man with newly diagnosed non-Hodgkin lymphoma. Top row images were acquired with GE Discovery 600 and reconstructed with OSEM. A left pleural-based lesion is identified. Bottom row images were acquired immediately after with GE Discovery MI and reconstructed with BPL. The same left pleural-based lesion is noted; however, a small cardiophrenic lymph node is also seen (red arrow)

The superiority of SiPM-based PET/CT (GE Discovery MI, DMI) versus photomultiplier tubes (PMT)-based PET/CT (GE D600/D690) was shown in cancer patients undergoing ^18^F-FDG imaging using OSEM as a reconstruction method for both scanner systems (Fig. 4). At the time of that study, the DMI had not been approved by the Food and Drug Administration; therefore, the standard scanner was always used first, followed by SiPM-based PET/CT [40]. Additional work compared DMI and D690 by randomizing patients referred for ^68^Ga-DOTATATE and ^68^Ga-PSMA11 to be scanned first on D690 followed by DMI or vice versa. The SUV_max_ measurements were higher in lesions detected by SiPM than by conventional PET/CT, regardless of the order of the scan. There were lesions only identified using the SiPM PET/CT showing that SiPM PET/CT has superior performance compared to a conventional PET/CT scanner [41].

## Motion correction in PET – standing at the edge of a paradigm shift

Patient motion, such as due to respiration, degrades PET images. As PET technology improves, motion becomes an increasingly significant cause of artefact. Images are acquired over minutes, and both voluntary and involuntary motions of a patient introduce image blur. The task of motion correction is uniquely challenging – unlike other obstacles of resolution that the field has transcended, patient motion is very subject specific and causes image degradation to extents that vary widely in feature and magnitude. The last few years have seen exciting advancements in PET motion correction and have the potential for widespread availability and use of motion-corrected images. Specifically, data-driven motion correction (DDMC) and the emergence of artificial intelligence (AI) tools are opening new, exciting opportunities for motion handling in clinical PET as well as new avenues for research and development [42].

For contextual comparison, let us consider motion handling in external beam radiotherapy. The effects of patient motion during treatment planning, and delivery, are well documented, and motion correction has become a routine and expected aspect of treatment protocols at many centres [43]. The principles of correcting for motion to improve care in radiotherapy and diagnostic PET are the same. Static images most closely match the required representation of a patient and therefore have the most utility, when motion is removed and/or accounted for. It is therefore crucial for the diagnostic imaging community to strive to provide the same high standard of care through motion correction.

**Fig. 5:**
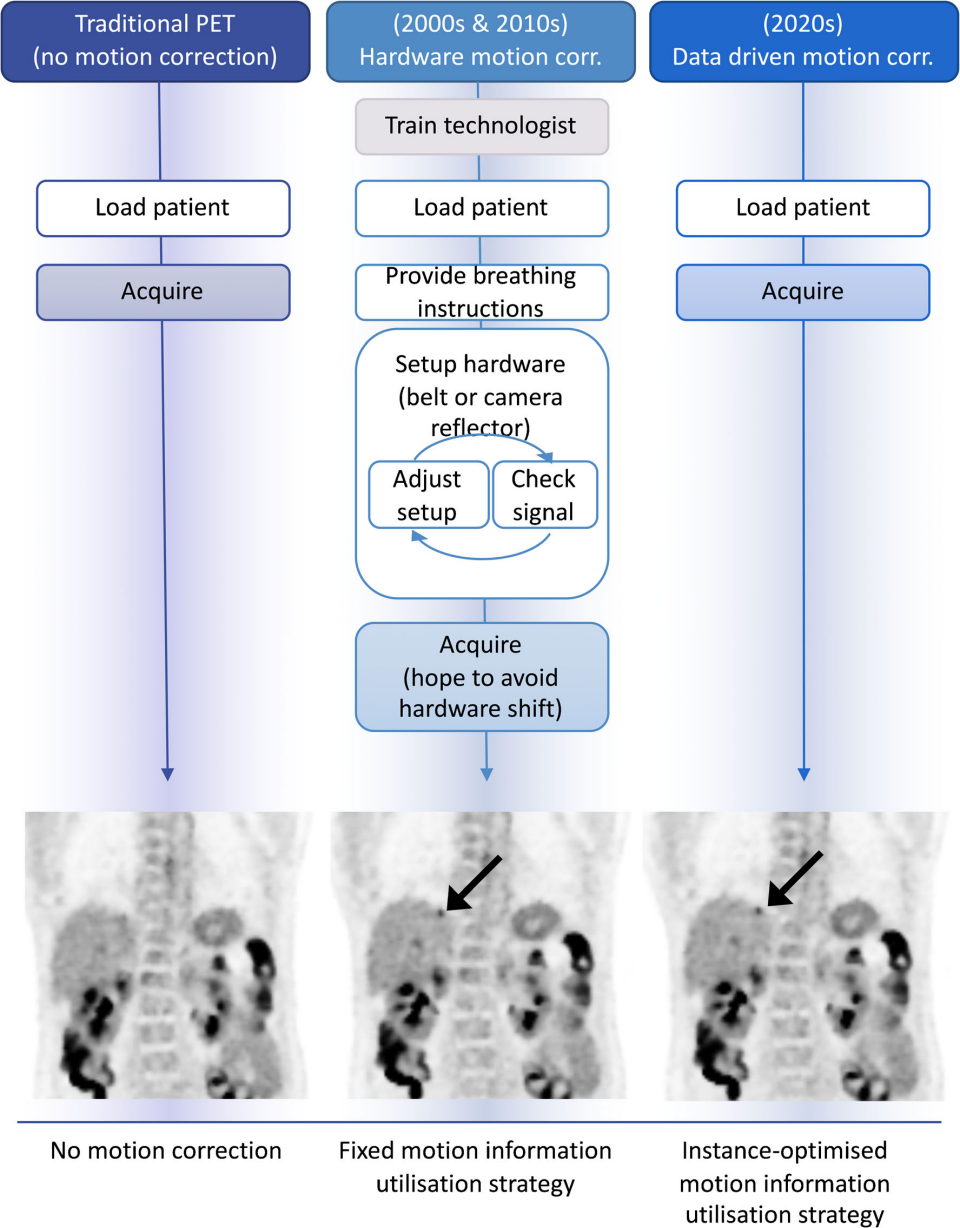
Improvements in motion correction. PET acquisition workflows shown for no motion correction, hardware-driven motion correction and data-driven motion correction. Images adapted from work originally published in JNM by Walker et al. [44]

Traditional motion correction solutions utilize hardware-based motion tracking during scan acquisition, most commonly a camera or pressure belt. DDMC is a more practical solution. DDMC offers hardware-free motion detection and correction, enabling fully automated patient benefits, while sparing users of complexity, variability, time, and staff radiation dose. The underlying concept of DDMC is to utilize existing information in the PET acquisition data, rather than an external signal, to characterize and correct for motion in that data. This information in the PET data has traditionally been ignored but is generated in every PET scan and is readily available through modern computer systems. An important feature of this technology is its practicality, which supports its easy implementation in the clinic as a default process, as well as supporting research. The easy DDMC workflow is demonstrated in Fig. 5.

DDMC has been developed and refined over the last 15 years [45]. The development of DDMC methods continues to be an active field of research [46–50]. Strategies have already been shown to match or outperform classical hardware-based technology in several studies [44, 51–54]. At present, and what makes this moment a pivotal point of transition, three major PET vendors now offer DDMC tool options on the new generations of their PET scanners: GE, Siemens and United Imaging (as declared on their websites). Notably, the vendor tools provide fully automated workflows that culminate with a ‘simple’ final 3D motion-corrected image. Using these tools, we are already seeing a new research arena with large patient cohorts being studied [44, 46, 51, 55], providing statistical power to demonstrate a desirable evolution of quantitative and qualitative PET through the use of DDMC. Encouragingly, we are also seeing research studies branching out beyond the traditional PET motion correction focus of lung lesion quantification to address additional clinical applications, such as cardiac imaging and diagnostic tumour/organ differentiation [47, 48, 55, 56].

Benefits from DDMC promise to advance, at least incrementally, all areas of PET that rely on quantification and resolution in anatomy subject to motion, areas that are not able to reap the full benefits of current scanner resolutions. Better imaging will mean better detection, uptake classification, treatment monitoring, radiomics, segmentation and dosimetry. Ultimately advancements across these areas will support real improvements for, and evolution of, patient care.

In summary, existing techniques and newly available commercial technology can reasonably support a transition in PET imaging to embrace motion correction as a new standard of care. This would match previous innovation transitions such as ToF, CT co-registration, 3D acquisition and iterative reconstruction, which we use by default – technology advancements that most of us cannot imagine being without! As a community, we need to document the features and drawbacks of DDMC technology so that clinicians can make informed decisions on whether they can support changes in their practice. Looking ahead, we can anticipate that future advancements in PET hardware, and DDMC software, will further enhance clinical PET to the benefit of all of our patients.

## Total-body PET

Anybody involved in designing or analysing PET studies recognizes that limited detection sensitivity, and its consequence of image noise, is by far the biggest technical limitation in PET imaging, whether that be in clinical PET or in research studies. PET images are routinely reconstructed and processed at a spatial resolution far worse than the underlying detector technology is capable of, and scan times are relatively long, which then causes patient motion to further degrade spatial resolution. In addition, the administered dose required for acceptable image quality limits PET to a fairly narrow range of clinical applications that represent a mere fraction of the potential afforded by the exquisite specificity and sensitivity of the radiotracer method. Furthermore, clinical PET has never been able to exploit the strengths of dynamic imaging and kinetic modelling, in part because the signal-to-noise is not good enough, and becomes even worse when attempting to dynamically cover a large fraction of the body by using multiple passes through the body which leads to poor temporal sampling at any one location.

**Fig. 6:**
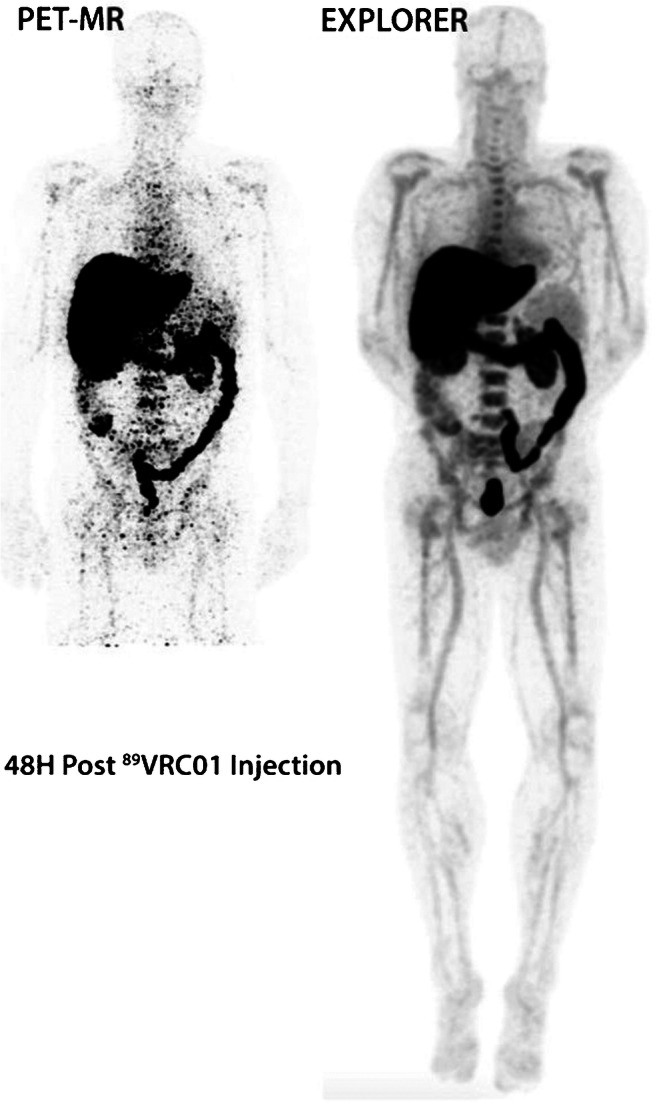
Images acquired from the same subject for 48 mins on conventional PET/MR (left) and for 20-min total-body PET (right). Radiotracer: 33 MBq ^89^Zr-VRC01. Time after injection: 48–52 h. Images adapted from work originally published in JNM by Beckford Vera et al. [62]

Total-body PET is a transformative technology that changes the equation [57–61]. The use of detectors that cover the entire human body not only increases the imaging field of view but, by increasing the solid angle coverage, increases detection efficiency, such that for a given administered dose and acquisition time, roughly 40-fold more events are collected from the whole body. This step change in performance opens up a vast new parameter space for PET, which at its extremes permits imaging at up to 6-fold higher signal-to-noise levels (e.g. Fig. 6), effective radiation doses in the range of that received for a round-trip transatlantic flight, or imaging of the entire body in a minute or less [57]. Crucially, it not only, like previous advances in PET technology, allows us to do things we currently do but better; it also allows us to do things we have never been able to do before. Specifically, the ability to image radiotracer kinetics in every tissue and organ of the body simultaneously, with absolute quantification provided non-invasively from an image-derived arterial input function that always is available within the field of view. Thus total-body PET enables new science, and the chances are high this will at some time in the future translate to new clinical applications.

What is not to like about a technology that by definition addresses the most significant weakness in PET and nuclear medicine and improves EVERY study, either by improving the signal-to-noise ratio, reducing the dose, reducing the scan time or some combination of the three [63]. Of course, the adoption of such systems entails a significant learning curve. The step change in performance raises many questions about optimal protocol design and the relative importance of image quality, scan time and injected dose, all of which should be evaluated in the context of the task at hand. But it would be a terrible waste if total-body PET was just to scan faster or at a lower dose where that is not necessary. The power of total-body PET is that it can provide superior quality kinetic data across the entire body, and that is where the focus should be.

Total-body PET is also an opportunity to revisit old ideas and dogma. Some radiotracers that were previously discarded perhaps deserve re-evaluation on a system with much higher temporal resolution or signal-to-noise capabilities. In clinical care, optimal uptake times need reassessment and should be aligned with the capabilities of these new systems as well as the underlying biology and kinetics of the radiotracer.

Total-body PET is disruptive not just because it is a technical game changer but also because it challenges the status and economics of PET. Nuclear medicine is a medical imaging niche that historically has been a poor driver of revenue. As a result, we have spent so long at a table where major investments in nuclear medicine have been judged unjustified, that we have come to agree with such judgements. Some of us in nuclear medicine seem to be suffering from collective ‘Stockholm syndrome’ where we sympathize with, and justify the arguments of, the naysayers in our institutions. It took 10 years of grant-seeking culminating in a $15 m award to create the first total-body PET scanner for humans. In the prior decade, investments twice this size were made to build advanced MRI scanners, while barely generating news coverage. MRI protocols routinely employ imaging times comparable to those required for parametric PET imaging, yet we tell ourselves that dynamic PET imaging is ‘not practical for the clinic’.

We should ask what the price is of *not* embracing technologies and methods that move the field forwards with large performance gains that open up a broad range of new applications. Without innovation at all levels, including the technology, radiotracers, algorithms and applications, our field will stagnate, and we will not realize the full potential of the radiotracer method in research or clinical applications. We will also fail to attract new talents to our field that are excited and have a vision for developing the new applications enabled by technological breakthroughs such as total-body PET. Ultimately, it would be a disservice to our patients who are depending on us to deliver imaging solutions that can better inform their diagnosis and direct their treatment.

Of course, total-body PET ultimately must demonstrate that the benefits justify its cost, but it can only do that if the technology is first developed and then disseminated to create a critical mass of centres and expertise that can generate the data which will ultimately answer that question. Without taking bold steps, the field will never progress more than incrementally. We believe total-body PET serves as a reminder of the power of the radiotracer method and is evidence that today’s systems still can and must evolve to take full advantage of the strengths of nuclear medicine. Next generation total-body systems, through major improvements in time-of-flight resolution, along with an intelligent application of deep-learning approaches promise another step change in performance, and so the innovations continue.

## Artificial intelligence–based image enhancement

Despite the regular increase in detection sensitivity of PET scanners, in particular thanks to the longer axial field of view, PET images are affected by noise, which complicates the interpretation of small regions with suspicious elevated uptake. New deep learning algorithms are now described and commercially available to convert noisy PET images into high-quality noise-free images. The algorithms can be trained using pairs of high-noise/low-noise images, where the high-noise image can be obtained by reconstructing only a fraction of the events detected during a complete acquisition and the low-noise image is the image corresponding to the complete acquisition yielding diagnostic image quality. Alternatively, the pair of high-noise/low-noise images could be obtained using realistic simulations. The network then learns the non-linear transformation that can convert a high-noise image into a low-noise image, based on all examples used for training. The training can be based on the high-noise/low-noise PET image pairs only [64] or can also take advantage of anatomical information provided by CT or MR images [65, 66]. In that latter case, the low-noise scan is learnt based on the high-noise scan and the anatomical information, which has been shown to improve the recovery of structural details in the PET images. In that AI application, the deep network acts as an optimal non-linear filter, learned based on many examples and integrating anatomical priors when the CT and MR scan is used. Filters have been used for ages in image reconstruction, first associated with filtered back projection and then with iterative reconstruction, to compensate for excessive noise associated with the restoration of high-frequency signals. Advances in AI now offer us access to sophisticated non-linear filters that may more easily account for the structural information, without laborious and subjective fine-tuning of hyperparameters, weighting the relative attachment of the reconstructed image to the measured data and to the anatomical priors. Although thorough evaluation studies are absolutely needed to extensively characterize the impact of such AI-based filtering on lesion detection and on quantitative values measured from the images, the availability of such AI-based image enhancement should be seen as another opportunity to further improve image quality obtained for a given injected dose and scan time. In addition, only a broad assessment of these new image-enhancement approaches based on machine learning will make it possible to collect more data, including outlier examples, needed to make the methods evolve and become more and more accurate and robust. Clinical availability and assessment are thus key steps for making these appealing methods progress towards bringing indisputable benefit to patients. If proven mostly unbiased, these algorithms could of course also be seen as a way to get clinically interpretable images with less dose [67] or with shorter acquisition times. Yet, the first priority should be to deliver the best possible image quality for ensuring the highest-quality report.

## AI-based abnormality detection

AI also holds great promise to assist in image interpretation. AI-based prototypes are now available to assist in the identification of suspicious high-uptake regions in FDG PET/CT scans and accelerate the associated reporting [68]. Automated identification is based on the training of the algorithm from a large number of PET/CT scans acquired in lymphoma and non-small cell lung cancer patients thoroughly labelled by experts. Based on the features and location of the elevated uptake in the many examples used for training, the algorithm learns to distinguish physiological uptake from so-called suspicious foci. The result is presented to the user for further analysis, where the user can remove regions that have incorrectly been labelled as suspicious or add high-uptake regions that were missed by the AI. Based on the set of resulting ‘suspicious’ regions, the total metabolically active tumour volume can be automatically calculated and reported [69]. As in any algorithm relying on training, the performance of the system depends on the variety of the scans used for training and on their representability compared to the scans that the algorithm will be exposed to. Very promising results have been reported in lymphoma patients, both regarding the ability of the algorithm to identify lesions labelled by an expert in a broad range of scans acquired with a large variety of imaging systems and for estimating a total tumour volume with similar prognostic value as when this volume is calculated by experts [69]. These results suggest that the algorithm performs well even on images acquired on a scanner that was not the one used to produce the training images. In addition, the algorithm appeared to also yield encouraging results in patients with advanced breast cancer, while no breast cancer patients were used to train the algorithm, suggesting that the algorithm can identify suspicious uptake associated to a disease that was not represented in the training set [70]. Yet, some small cancer foci were missed, which demonstrates the need to further improve the AI-based algorithm. Similar to all algorithms based on machine learning, comprehensive tests of the prototype will be needed to identify cases where the algorithm fails and introduce such cases in the training set so as to improve the algorithm performance. Hence, in all machine learning applications likely to assist the user, availability and use are essential to further develop and improve the algorithms. Availability for clinical assessment does not imply clinical adoption, and only the results of sound evaluation studies performed by independent groups will tell whether the technology is mature enough and can be adopted. Still, such algorithms should be given a chance to contribute to the unavoidable reshaping of the medical image interpretation associated with the advent of AI in radiology.

## Discussion

### How fast can or should we scan patients using modern PET systems?

An issue faced by PET unit teams is the management of patients experiencing pain or dyspnoea. Also challenging is scan acquisition in uncompliant paediatric patients (when premedication is not efficient or feasible) and claustrophobic patients. Positioning the patient on the table (especially for bedridden patients) and PET acquisition are the bottleneck of the total scan time (with the exception of total-body PET). Therefore, the fastest PET acquisition would be very useful in all of these situations, provided the diagnostic performances are maintained. Fast PET acquisition should be used for specific populations likely to benefit from fast or ultra-fast imaging, and not for economic reasons, though improving the throughput of a PET unit may be considered useful to reduce waiting time for urgent PET scans.

**Fig. 7:**
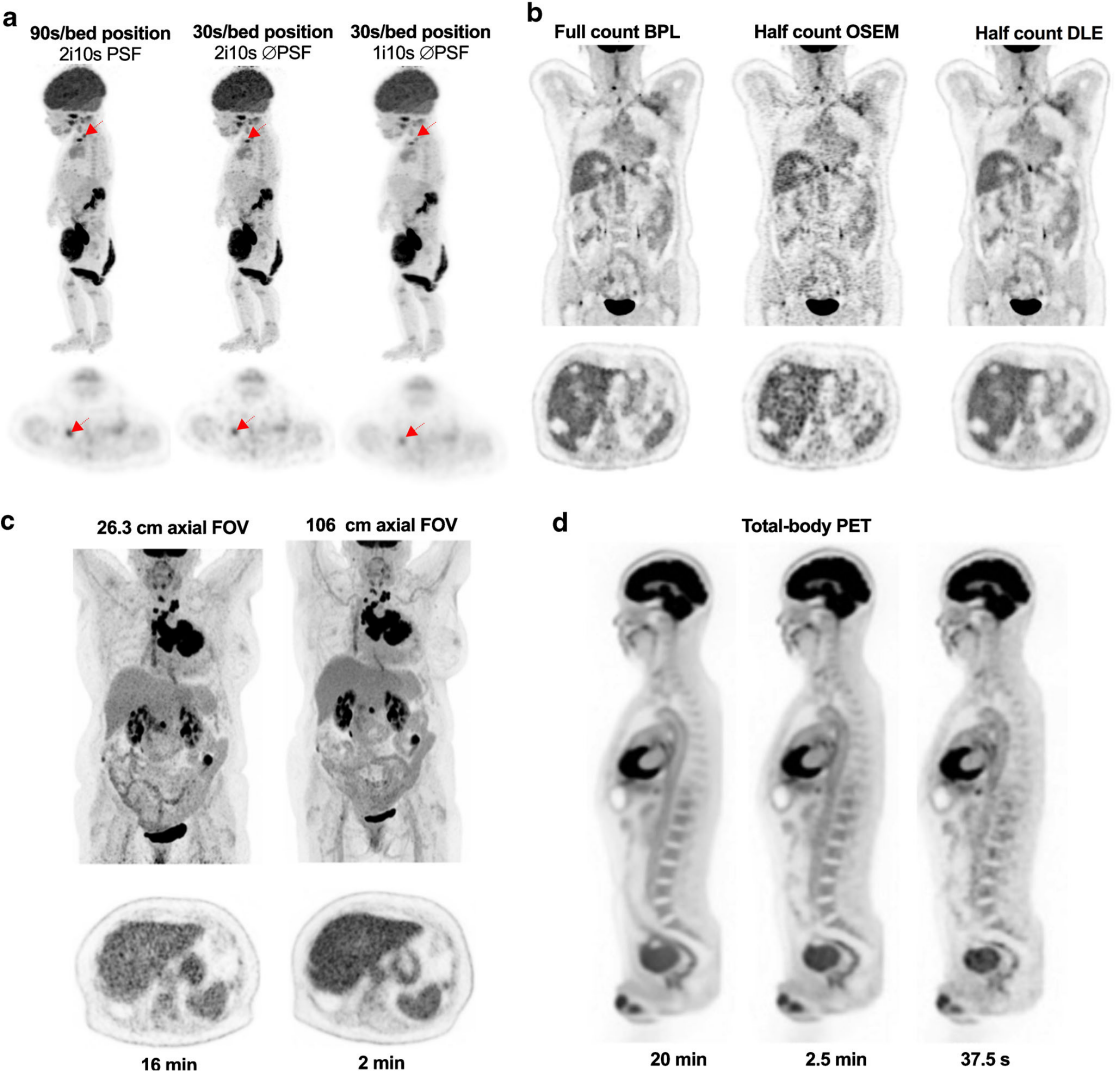
From fast to ultra-fast PET imaging using SiPM PET (**a**), AI-based denoising (**b**), PET systems with long axial FOV (**c**) and total-body PET (**d**). Panel **a** shows that SiPM PET demonstrated that acquisition time per bed position on the Vereos system can be reduced from 90 s provided reconstruction parameters are optimized (here by decreasing the number of iteration and disabling PSF modelling). Panel **b** displays an example of fast imaging using an AI algorithm: it is possible to reconstruct data from half the available counts on an OSEM image and get image quality equivalent to a full count BPL image using a deep learning enhancement algorithm [71]. Panel **c** (adapted from [72]): equivalent signal-to-noise ratio and subjective image quality can be achieved using a 2-min acquisition on the Siemens long axial FOV SiPM, compared to a standard acquisition on the standard axial field-of-view Biograph Vision 600. Panel **d** (adapted from [63]): images of varying scan duration using the EXPLORER total-body PET scanner (290 MBq ^18^F-FDG injected, 82-min uptake period). The apparent noise increases as scan time is decreased, but the images appear to be of diagnostic quality even at 37.5 s

Several options are possible to achieve fast or even ultra-fast PET imaging (Fig. 7):

- Modern PET systems allow a significant reduction of scan time, provided reconstruction parameters are optimized to reduce noise in the images. This can be achieved using a standard injected dose, as shown by Sonni et al. with the GE Discovery MI [73] who showed 1 min/bed images were of equal quality to 3–4 min/bed, or by increasing dose, as shown by Coudrais et al. [74] in a non-inferiority study seeking the fastest acquisition time possible with the Phillips Vereos system. This study demonstrated that acquisition time per bed position on the Vereos system can be reduced from 90 to 30 s without significant impact on quantitative and visual image quality and with preservation of a good detectability as compared to the standard reconstruction. These fast acquisitions required the optimization of reconstruction parameters. Similar results were obtained on the Siemens Vision system [75, 76]. With some low-count images, it may be necessary to turn off PSF modelling, but where BPL reconstruction is used, a high regularizing term can be used to counteract the noise in the image due to the low number of counts.
- In the context of fast PET imaging, denoising of PET images acquired with low counts can be achieved using AI-based reconstruction. A commercial supplier has investigated this and has developed an algorithm that could be used for halving the injected activity or scan time by using an AI algorithm to convert half count OSEM images to full count BPL images [71]. An academic group has also investigated this using an eighth of the injected activity or scan time [77]. Future work in this area is likely to bring significant patient benefit and enable further increase in scanning speed or decrease in injected activity (and so patient and staff radiation dose).
- Through the huge increase in sensitivity given by total body PET, one application (for specific patients) is to inject patients with the ‘standard’ activity and allow truly ultra-fast imaging of them.

### The issue of harmonization

There are two points of view regarding harmonization: first, that the best-quality images available should be used for interpretation, in order to avoid a lowest common denominator image quality which fails to do the best for our patients, or the other view, to only use harmonized images such as those approved by EARL [14] to ensure consistency between scanners and sites. A compromise is proposed whereby the best reconstruction possible (such as using PSF or BPL) is used for lesion detection, but in the case of multicentre trials, or where images need to be compared from old to new cameras, then harmonized metrics can be used in addition specifically for this purpose [78–81]. The quantitative values displayed in Fig. 3b and d would fit into the updated EARL standards, the so-called EARL2 [82, 83], chosen to meet the standards of modern PET scanners. Where at all possible sites, should be encouraged to store their PET sinograms, this would enable older PET images to be re-reconstructed with the latest algorithm, for the specific case where the newer technology uses an updated image reconstruction such as including PSF modelling or BPL regularization, which would aid the reporter for comparisons. This is obviously not possible where the scanner itself is upgraded to SiPM detectors (or total body) as it is infeasible to improve the sensitivity or resolution of previously collected PET images from the sinograms! Harmonization of measured values, such as SUV_max_ or metabolically active tumour volume, is also possible to pool data acquired using different scanners and protocols to perform multicentre studies while taking advantage of the best image quality each scanner can offer [80, 84].

### Evidence base

Adequate scientific evaluation of these technologies is crucial, often through fruitful partnerships between users and PET vendors: none of these technologies should be considered erroneous until proven otherwise. Evolution and developments are generally positive for the field and crucially are very much in the best interests of patients. As medical professionals, we should promote anything that can improve our patient’s care. Technological advances require early adopters to test out the technology and papers resulting from their use covering the benefits and disadvantages should be encouraged to be published so that all can learn. The initial papers on the technology can then be used by other sites to justify moving to using new technology.

## Should we revisit interpretation criteria?

### Deauville score

As shown in the study from Enilorac [12] and clearly confirmed in Fig. 1, which is, to the best of our knowledge, the largest series of PET examinations comparing PSF and conventional OSEM for risk stratification in DLBCL patients, the modelling of PSF within the reconstruction is not an issue in routine clinical processes or in multicentre trials. There is insufficient evidence to support the recommendation to not use more sensitive PET/CT reconstruction methods.

Based on the data presented in this manuscript (Fig. 1), most of the events occurring by 2 years in DS3 patients based on EARL-compliant reconstruction (60.0%) occurred in discordant DS4 patients with PSF reconstruction. It is plausible that these patients may have benefited from the implementation of PSF in clinical routine for the determination of their DS. Another important consideration is that PET technology is in constant development and rapidly evolving, the latest to date being SiPM PETs system and total-body PET. PET centres will surely want to benefit from the best capabilities of their new investments. It would be unheard of for a hospital to be rooted in technologies that are certainly pioneering but moving towards being obsolete or installing state-of-the-art equipment only to disable the leading edge components that are designed to maximize patient benefit. For example, no hospital would use a FBP reconstruction on a modern PET for clinical patients.

### Solid tumours: visual interpretation, SUVs and metabolically active tumour volume

There is obviously a learning curve to be observed when upgrading a PET system to a state-of-the-art system, whether it is hardware or software advancement. However, this does not mean these advanced methods should be avoided: we need to embrace technological innovation even if this lies outside our comfort zone. All subjects undergoing PET and specifically, cancer patients, should be staged and followed up as accurately as possible using the most sensitive technique available. While it is already known that there is no magic threshold when using SUV for diagnostic purposes, it is clear that new PET systems significantly increase SUV metrics, particularly for small foci, compared to former generation PET systems (Fig. 3). This may alter also the computation of the metabolically active tumour volume when using threshold-based contouring methods: volumes tend to be smaller when using advanced reconstruction methods compared to OSEM [82, 85]. Again, harmonization programs are useful to manage these issues, but despite being a great success, it is noteworthy that the number of EARL-accredited centres (250 at the time of the writing of this manuscript) is far inferior to the installed base of PET systems.

## Conclusion

The relatively rapid evolution in scanner technology, including SiPM PET/CT systems, promises further advances in image quality, gains in spatial resolution and further gains from time of flight, which when combined with modern image reconstruction methods, motion correction and AI-driven algorithms, will lead to yet more improvement in disease detection and quantification. Clinicians should be keen to adopt all of these into clinical practice as to do otherwise is not doing the best we can for our patients. We look forward to all users embracing technology, now and in the future, for improved early diagnosis and detection and crucially the benefit of patient care.
